# Spinal Obstruction-Related vs. Craniocervical Junction-Related Syringomyelia: A Comparative Study

**DOI:** 10.3389/fneur.2022.900441

**Published:** 2022-08-01

**Authors:** Chenghua Yuan, Jian Guan, Yueqi Du, Zeyu Fang, Xinyu Wang, Qingyu Yao, Can Zhang, Shanhang Jia, Zhenlei Liu, Kai Wang, Wanru Duan, Xingwen Wang, Zuowei Wang, Hao Wu, Zan Chen, Fengzeng Jian

**Affiliations:** ^1^Department of Neurosurgery, Xuanwu Hospital, Capital Medical University, Beijing, China; ^2^Spine Center, China International Neuroscience Institute (CHINA-INI), Beijing, China; ^3^Laboratory of Spinal Cord Injury and Functional Reconstruction, Xuanwu Hospital, Capital Medical University, Beijing, China; ^4^Research Center of Spine and Spinal Cord, Beijing Institute of Brain Disorders, Capital Medical University, Beijing, China; ^5^National Center for Neurological Disorders, Beijing, China

**Keywords:** syringomyelia, decompression, spinal cord injury, biomarker, CSF

## Abstract

**Background:**

No prior reports have focused on spinal cord injury (SCI) characteristics or inflammation after destruction of the blood–spinal cord barrier by syringomyelia. This study aimed to determine the differences in syringomyelia-related central SCI between craniocervical junction (CCJ) syringomyelia and post-traumatic syringomyelia (PTS) before and after decompression.

**Methods:**

In all, 106 CCJ, 26 CCJ revision and 15 PTS patients (mean history of symptoms, 71.5 ± 94.3, 88.9 ± 85.5, and 32.3 ± 48.9 months) between 2015 and 2019 were included. The symptom course was analyzed with the American Spinal Injury Association ASIA and Klekamp–Samii scoring systems, and neurological changes were analyzed by the Kaplan–Meier statistics. The mean follow-up was 20.7 ± 6.2, 21.7 ± 8.8, and 34.8 ± 19.4 months.

**Results:**

The interval after injury was longer in the PTS group, but the natural history of syringomyelia was shorter (*p* = 0.0004 and 0.0173, respectively). The initial symptom was usually paraesthesia (*p* = 0.258), and the other main symptoms were hypoesthesia (*p* = 0.006) and abnormal muscle strength (*p* = 0.004), gait (*p* < 0.0001), and urination (*p* < 0.0001). SCI associated with PTS was more severe than that associated with the CCJ (*p* = 0.003). The cavities in the PTS group were primarily located at the thoracolumbar level, while those in the CCJ group were located at the cervical-thoracic segment at the CCJ. The syrinx/cord ratio of the PTS group was more than 75% (*p* = 0.009), and the intradural adhesions tended to be more severe (*p* < 0.0001). However, there were no significant differences in long-term clinical efficacy or peripheral blood inflammation markers (PBIMs) except for the red blood cell (RBC) count (*p* = 0.042).

**Conclusion:**

PTS tends to progress faster than CCJ-related syringomyelia. Except for the RBC count, PBIMs showed no value in distinguishing the two forms of syringomyelia. The predictive value of the neutrophil-to-lymphocyte ratio for syringomyelia-related inflammation was negative except in the acute phase.

## Introduction

The most common clinical manifestation of syringomyelia is dilatation of the central canal of the spinal cord. It is often associated with Chiari malformation (CM), basilar invagination or atlantoaxial dislocation, arachnoid adhesion, other cerebrospinal fluid circulation disorders (1–6), and other spinal deformities. Thus, it is a type of chronic central spinal cord injury (SCI) (7). Approximately 20% of patients with SCI caused by trauma have syringomyelia (8, 9). In some cases, the cavity can be reduced by intradural decompression (10). However, SCI-related symptoms, such as dissociative sensory disturbances, muscle atrophy, and joint deformity, are often unimproved and can even worsen (1). Therefore, repairing the SCI caused by the cavity is a bottleneck in clinical treatment. Ependymal cells surrounding the central canal are a source of endogenous stem cells, indicating a potential method for endogenous SCI repair (11, 12).

To date, no feasible non-canine animal models of CM have been established except compression models (13, 14). SCI is accompanied not only by damage to the nerve tissue–cerebrospinal fluid barrier but also by damage to the nerve tissue–blood barrier (15). Therefore, the pro-oxidation and antioxidation processes that occur in the central nervous system (CNS) may be reflected in the components of the cerebrospinal fluid (CSF) and blood. A better understanding of the potential molecular pathways associated with syringomyelia formation will reveal targets for the treatment and prevention of syringomyelia.

Therefore, we performed a retrospective review of our institutional cases of different pathological circumstances of syringomyelia, i.e., craniocervical *vs*. spinal CSF obstruction, and compared them in terms of natural history, symptom presentation and progression, and blood biomarkers and outcomes.

## Methods

The study was reviewed and approved by the local ethics committee; the requirement for informed consent from patients was waived given the retrospective nature of the study.

**Figure 1:**
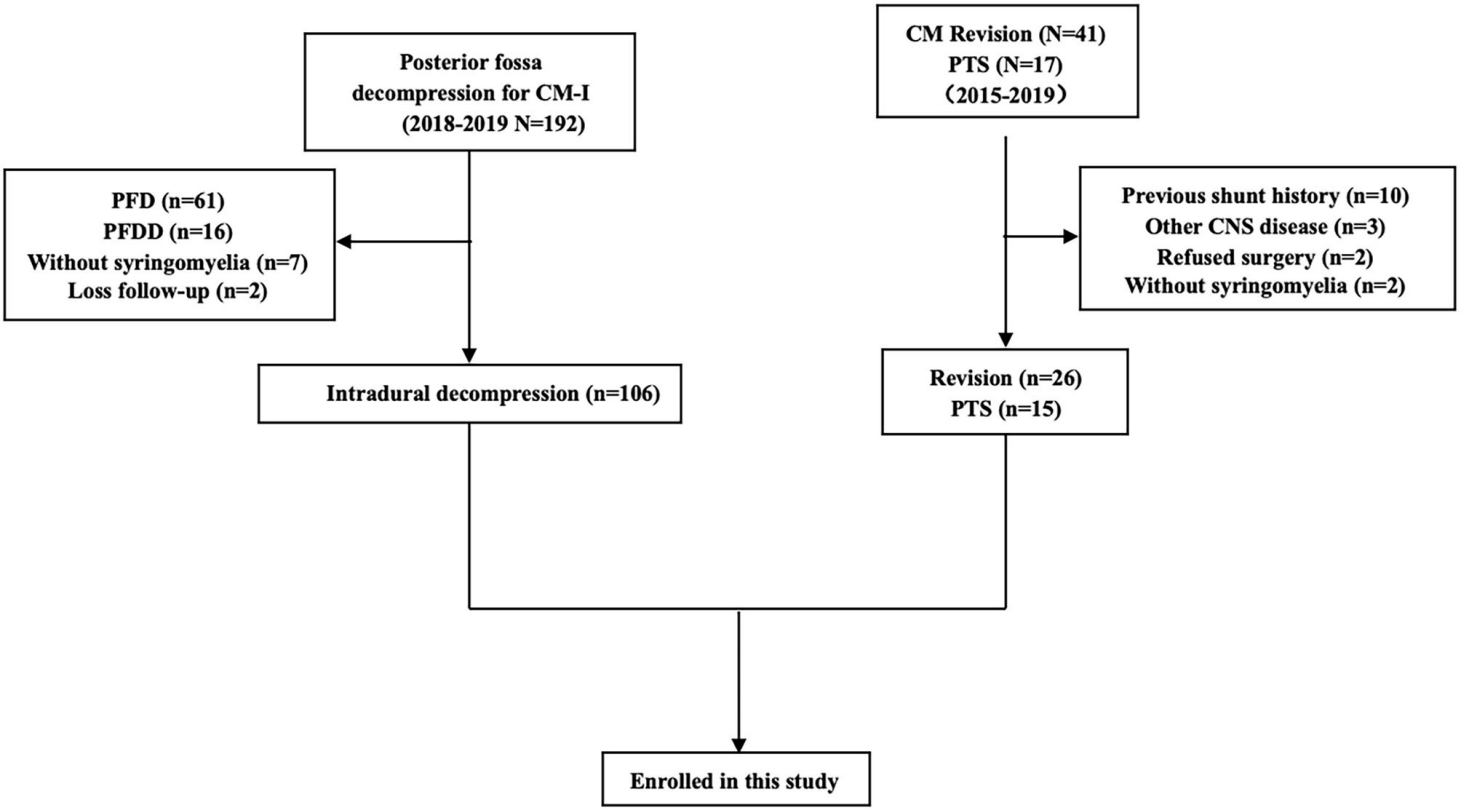
Flow chart of 147 consecutive syringomyelia patients between 2015 and 2019.

Between January 2015 and December 2019, there were 146 consecutive patients treated with intradural decompression for syringomyelia associated with CM, treated with revision or treated for post-traumatic syringomyelia (PTS) at our institution (Table 1). In this study, PTS was defined as local arachnoid obstruction. The detailed inclusion and exclusion criteria are shown in Figure 1.

**Table 1:** Perioperative clinical data of patients with syringomyelia of different etiologies.

	**Chiari I malformation** **(*n* = 106)**	**Revision** **(*n* = 26)**	**PTS** **(*n* = 15)**	***P***
**Male**	25(23.58%)	7(26.92%)	13(86.67%)	**<0.0001** ^**a**^
**Age, years**	48.0 ± 12.7	47.0 ± 11.3	50.5 ± 8.4	0.8018^b^
1–20	5(4.71%)	0	0	
20–29	3(2.83%)	3(11.54%)	0	
30–39	14(13.21%)	4(15.38%)	1(6.67%)	
40–49	29(27.36%)	5(19.23%)	6(40%)	
50–59	36(33.96%)	11(42.31%)	5(33.33%)	
60+	19(17.92%)	3(11.54%)	3(20%)	
**Previous ASIA**				**0.001** ^**c**^
Complete	NA	0	6(40%)	
Incomplete		26(100%)	9(60%)	
**Previous surgery**				
Yes	NA	26(100%)	9(60%)	
Conservative		0	6(40%)	
**Interval**	NA	61.7 ± 60.4	203.0 ± 136.4	**0.0004** ^d^
**SM Symptom duration, months**	71.5 ± 94.3	88.9 ± 85.5	32.3 ± 48.9	**0.0173** ^b^
<1	4(4.08%)	0	1(6.67%)	
1–6	22(22.45%)	1(3.85%)	4(26.67%)	
7–24	25(25.51%)	6(23.08%)	7(46.66%)	
>24	47(47.96%)	19(73.07%)	3(20%)	
**First SM sign**				0.258^c^
Neuropathic pain	32(32.65%)	11(42.31%)	2(13.33%)	
Dysesthesia	37(37.75%)	11(42.31%)	9(60%)	
Sensory deficit	9(9.18%)	0	0	
Motor	20(20.42%)	4(15.38%)	4(26.67%)	
**Symptoms**				
Occipital pain	31(29.24%)	12 (46.15%)	0	0.099^a^
Neuropathic pain	59(55.66%)	19 (73.08%)	7(46.67%)	0.178^a^
Dysesthesia	75(70.75%)	22(84.62%)	12(80%)	0.303^a^
Hypesthesia	61(57.55%)	22(84.62%)	13(86.67%)	**0.006** ^**a**^
Motor power	50(47.17%)	18(69.23%)	13(86.67%)	**0.004** ^**a**^
Gait	31(29.24%)	14(53.85%)	13(86.67%)	**<0.0001** ^**a**^
Sphincter function	9(8.49%)	3(11.54%)	10(60%)	**<0.0001** ^**c**^
Swallowing function	11(10.38%)	9(34.62%)	2(13.33%)	**0.01** ^**c**^
Sweating	14(13.21%)	2(7.69%)	2(13.33%)	0.844^c^
**ASIA**				
UE	48.2 ± 5.4	47.7 ± 2.8	47.7 ± 3.5	0.1012^b^
LE	48.8 ± 5.4	48.3 ± 3.3	39.9 ± 14.2	**<0.0001** ^**b**^
PP	104.5 ± 12.2	105.3 ± 7.9	91.5 ± 11.5	**0.0005** ^**b**^
LT	106.1 ± 11.4	105.3 ± 7.8	94.0 ± 10.1	**<0.0001** ^**b**^
A	0	0	0	**0.003** ^**b**^
B	1(0.94%)	0	1(6.67%)	
C	1(0.94%)	0	4(26.67%)	
D	90(84.91%)	25(96.15%)	9(60%)	
E	14(13.21%)	1(3.85%)	1(6.67%)	
**Radiological data**				
Ventilation dilation	7(6.60%)	1(3.85%)	0	0.945^a1^
Scoliosis	22(20.75%)	5 (19.23%)	3(13.33%)	0.984^a^
Occipitalization of atlas	8(7.55%)	3(11.54%)	0	0.792^a1^
Basilar invagination	12(11.32%)	5(19.23%)	0	0.452^a1^
Klippel-Feil syndrome	3(2.83%)	0	0	1.00^c^
Syringomyelia	106(100%)	26(100%)	15(100%)	
**Lesion location**				
Medulla oblongata	2(1.89%)	0	2(13.33%)	
Cervical	14(13.21%)	3 (11.54%)	0	
Cervicothoracic	90(84.91%)	23(88.46%)	8(46.67%)	
Thoracic	1(0.94%)	0	2(13.33%)	
Thoracolumbar	0	0	1(6.67%)	
CTL	1(0.94%)	0	4(20%)	
Whole	0	0	0	
**Cord/canal**				**0.009** ^**b**^
A	51(48.11%)	19(73.08%)	11(73.34%)	
B	24(22.64%)	6(23.08%)	2(13.33%)	
C	26(24.53%)	1(3.84%)	2(13.33%)	
D	5(4.72%)	0	0	
E	0	0	0	
**Management**				
Arachnoid opened	106(100%)	26(100%)		
Tonsil manipulated	61(57.55%)	18(69.23%)		0.276^a^
Fusion	NA	NA	4(26.67%)	
Arachnoid lysis			4(26.67%)	
Laminectomy			1(6.67%)	
Arachnoid lysis+Syringostomy			1(6.67%)	
Arachnoid lysis+Cord transection			3(20%)	
Cord transection			1(6.67%)	
SSS			1(6.67%)	
**Intradural findings**				**<0.0001** ^**b**^
0	14(13.21%)	2(7.69%)	2(16.67%)	
1	89(83.96%)	19(73.08%)	0	
2	3(2.83%)	5(19.23%)	10(83.33%)	
**Complication**				0.133^c^
Isolated fever	2(1.89%)	0	1(6.67%)	
Aseptic meningitis	5(4.71%)	5(19.23%)	0	
CSF fistula	1(0.94%)	1(3.84%)	0	
Hydrocephalus	0	0	0	
Cerebral infarction	2(1.89%)	0	0	
Swallowing dys	0	0	0	
Haemorrhage	0	0	0	
Wound infection	0	0	0	
Urinary tract infection	0	0	0	
Pneumonia	0	0	1(6.67%)	
Total	10(9.43%)	6(23.07%)	2(13.33%)	
**Length of follow-up** (mos)	20.7 ± 6.2	21.7 ± 8.8	34.8 ± 19.4	

^*^Significant difference (p < 0.05) between groups.

*^a^Chi square test*.

*^a1^Correction of the Chi square test*.

*^b^Kruskal-Wallis test*.

*^c^Fisher exact test*.

*^d^Mann-Whitney test*.

*^e^One-way ANOVA test*.

**Figure 2:**
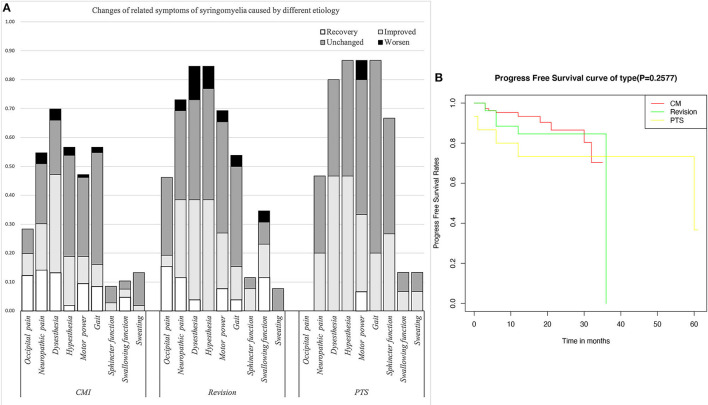
**(A)** Changes in related symptoms of syringomyelia of different etiologies. **(B)** Survival curve of syringomyelia.

**Figure 3:**
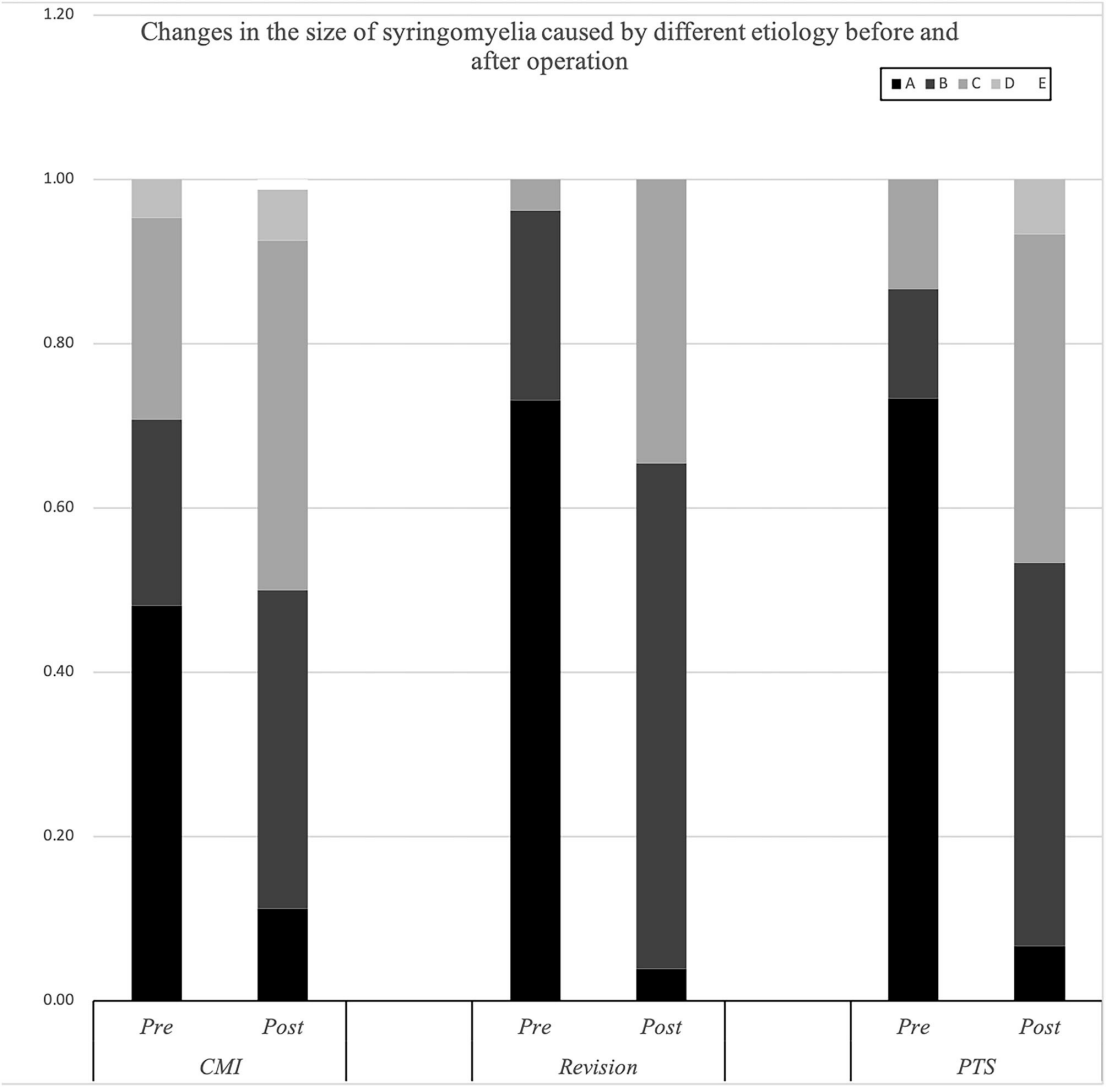
Changes in the size of the syrinx in syringomyelia of different etiologies before and after surgery.

**Figure 4:**
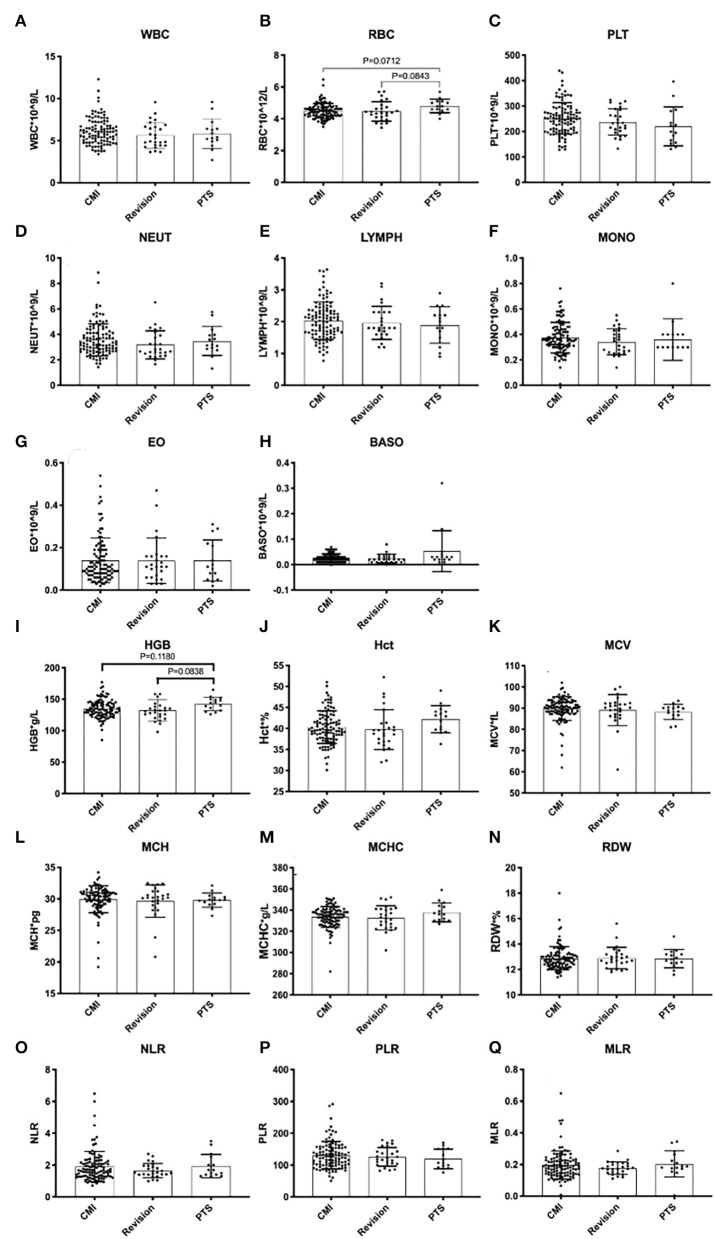
Peripheral blood inflammatory markers in syringomyelia of different etiologies.

The Klekamp–Samii (KS) score (16) and American Spinal Injury Association (ASIA) score (evaluated by YCH) were used to evaluate the clinical course of patients in the different groups before and after surgery. The long-term results are summarized with the Kaplan–Meier statistics (Figure 2). SC tension was defined as >75%, 50–75%, 25–50%, 10–25%, and <10% according to the syrinx/cord ratio (Figure 3). Peripheral blood inflammatory markers (PBIMs) were often tested 1 day before surgery (Figure 4).

**Figure 5:**
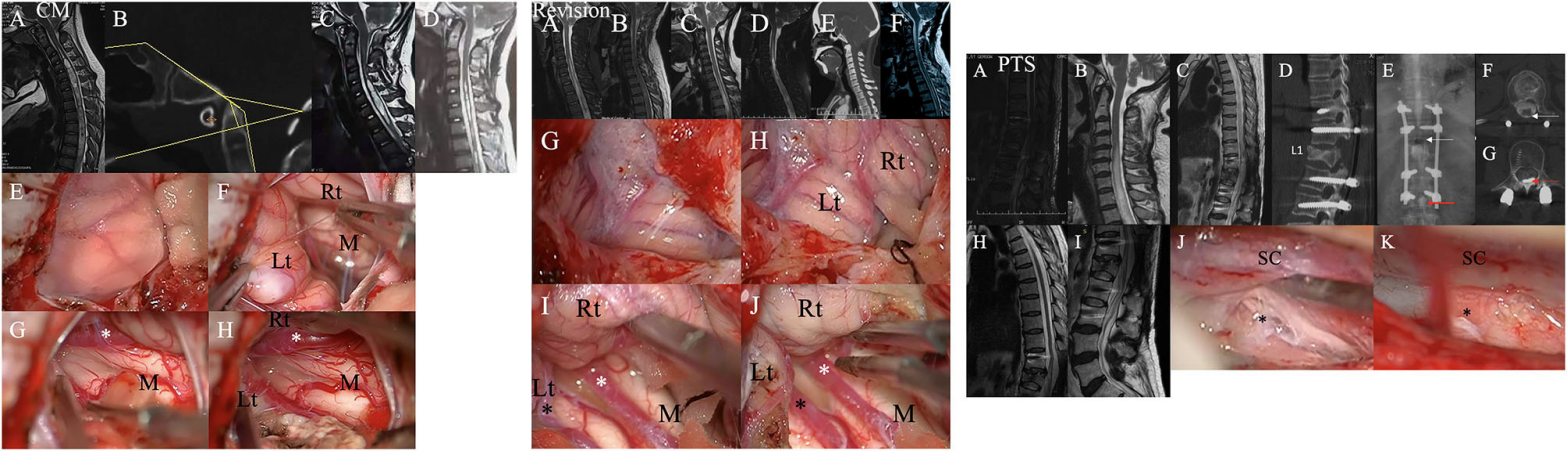
Left chart: Representative case from the CM group. A patient was found to have syringomyelia due to facial paralysis. **(A)** Schematic drawings of the foramen magnum region. Midsagittal T2-weighted MRI scans of the craniocervical region and CT scans suggested that there was no other instability or basilar invagination **(B)**. The ratio of the syrinx/canal from A to B three **(C)** months after the initial surgery and 30 **(D)** months after the initial surgery. **(E,F)** A hypertrophic tonsil obstructing the foramen of Magendie. **(G,H)** The right posterior inferior cerebellar artery (PICA) (asterisk) obstructing the foramen of Magendie was lysed, and the tonsil was coagulated. Lt, Left tonsil; Rt, Right tonsil; M, Medulla oblongata. Middle chart: Representative case from the revision group. **(A)** Preoperative sagittal T2-weighted MRI showed a large syrinx. **(B)** 3 months, **(C)** 9 months, and **(D)** 3 years after the first surgery. **(E)** CT showed partial bone defects of the occipital bone and persistent syringomyelia. **(F)** Postoperative MRI 2 years after the second surgery showing obvious reduction of the syringx. **(G,H)** A hypertrophic tonsil obstructing the foramen of Magendie. **(I,J)** The PICA (asterisk) obstructing the foramen of Magendie was lysed, and the tonsil was coagulated. Lt, Left tonsil; Rt, Right tonsil; M, Medulla oblongata. Right chart: Representative case from the PTS group. **(A)** Postoperative sagittal T2-weighted MRI scan showed some oedema and internal fixation. **(B–D)** Sagittal T2-weighted MRI and CT after 2 years showed a large syrinx (up to C4 and down to L1) and an L1 compression fracture. **(E–G)** Myelography showed that the circulation of CSF was blocked at L1. **(H,I)** Postoperative sagittal T2-weighted MRI data showed obvious reduction of the syrinx. **(J,K)**: Obvious adhesions around the spinal cord at L1 were removed intraoperatively.

Patients in the CM and revision groups required foramen magnum and foramen of Magendie dredging (FMMD), as previously reported (Figure 5) (10). According to the previous literature, the grade of intradural lesions was defined as 0, 1, or 2 (16). Finally, for PTS patients, we adopted anterior or posterior decompression. In addition, some authors have suggested an anatomy-based comprehensive classification of spinal osteotomies or arachnoid lysis (Figure 5) for compression fractures (7).

Follow-up data were obtained during outpatient visits or by telephone interviews. Treatment success was defined as a sustained improvement of preoperative symptoms or stabilization of previously progressing symptoms. Treatment failure was defined as postoperative neurological deterioration. Patients were assessed at 3 and 12 months postoperatively for neurological function using the KS score (Table 2). Long-term results in the three groups are summarized with the Kaplan–Meier statistics. The patients also underwent postoperative magnetic resonance imaging (MRI) to determine the tension of the syrinx.

**Table 2:** Changes in related symptoms of syringomyelia caused by different aetiologies.

**Symptom**	**Chiari I malformation** **(*n* = 106)**	**Revision** **(*n* = 26)**	**Craniocervical** **(*n* = 132)**	**PTS** **(*n* = 15)**
**Occipital pain**				
Preop	4.6 ± 0.7	4.4 ± 0.7	4.6 ± 0.7	0
Postop	4.7 ± 0.7	4.4 ± 0.7	4.6 ± 0.7	0
3 mos	4.7 ± 0.6	4.7 ± 0.6	4.7 ± 0.6	0
1 yr	4.8 ± 0.4	4.7 ± 0.5	4.8 ± 0.4	0
**Neuropathic pain**				
Preop	4.1 ± 0.9	3.7 ± 1.0	4.0 ± 0.9	3.9 ± 1.2
Postop	4.2 ± 0.9	3.8 ± 1.0	4.2 ± 0.9	4.1 ± 1.0
3 mos	4.4 ± 0.8	4.1 ± 0.8	4.4 ± 0.8	4.1 ± 1.0
1 yr	4.4 ± 0.8	4.0 ± 0.9	4.3 ± 0.8	4.1 ± 1.0
**Dysesthesia**				
Preop	3.5 ± 1.1	3.1 ± 1.0	3.4 ± 1.1	3.3 ± 1.1
Postop	3.9 ± 1.0	3.3 ± 1.1	3.8 ± 1.0	3.7 ± 1.0
3 mos	4.1 ± 1.0	3.4 ± 1.1	3.9 ± 1.0	3.7 ± 1.0
1 yr	4.1 ± 1.0	3.4 ± 1.1	3.9 ± 1.1	3.7 ± 1.0
**Hypesthesia**				
Pre-op	3.6 ± 1.3	2.7 ± 1.0	3.4 ± 1.3	3.0 ± 0.9
Post-op	3.6 ± 1.3	2.8 ± 1.0	3.5 ± 1.3	3.1 ± 0.7
3 mos	3.8 ± 1.2	2.9 ± 1.1	3.6 ± 1.2	3.3 ± 0.9
1 yr	3.8 ± 1.2	3.0 ± 1.1	3.6 ± 1.3	3.3 ± 0.9
**Motor weakness** ^**#**^				
Pre-op	4.3 ± 0.9	4.1 ± 0.8	4.2 ± 0.9	3.5 ± 1.1
Post-op	4.4 ± 0.8	4.2 ± 0.7	4.4 ± 0.8	3.6 ± 1.2
3 mos	4.5 ± 0.7	4.3 ± 0.7	4.5 ± 0.7	3.9 ± 1.1
1 yr	4.5 ± 0.8	4.3 ± 0.7	4.4 ± 0.8	4.0 ± 1.2
**Gait ataxia**				
Pre-op	4.5 ± 1.0	3.9 ± 1.1	4.4 ± 1.0	2.7 ± 1.5
Post-op	4.5 ± 0.9	4.0 ± 1.0	4.4 ± 1.0	2.5 ± 1.4
3 mos	4.6 ± 0.8	4.0 ± 1.1	4.5 ± 0.9	2.7 ± 1.4
1 yr	4.6 ± 0.8	4.2 ± 0.9	4.5 ± 0.8	2.7 ± 1.4
**Bladder function**				
Pre-op	4.8 ± 0.6	4.8 ± 0.6	4.8 ± 0.6	3.3 ± 1.4
Post-op	4.8 ± 0.6	4.8 ± 0.6	4.8 ± 0.6	3.3 ± 1.4
3 mos	4.8 ± 0.5	4.9 ± 0.4	4.9 ± 0.5	3.6 ± 1.3
1 yr	4.8 ± 0.5	4.9 ± 0.4	4.8 ± 0.5	3.6 ± 1.3
**Swallowing**				
Pre-op	4.8 ± 0.6	4.5 ± 0.7	4.8 ± 0.6	4.9 ± 0.3
Post-op	4.9 ± 0.3	4.8 ± 0.4	4.9 ± 0.4	4.9 ± 0.3
3 mos	4.9 ± 0.3	4.8 ± 0.5	4.9 ± 0.4	4.9 ± 0.2
1 yr	4.9 ± 0.3	4.7 ± 0.7	4.9 ± 0.4	4.9 ± 0.2
**Overall**				
Better	80(75.5%)	20(77.0%)	100(75.7%)	9(60.0%)
Unchanged	13(12.3%)	2(7.6%)	15(11.4%)	1(6.7%)
Worsen	13(12.2%)	4(15.4%)	17(12.9%)	5(33.3%)

^*^Unless otherwise specified, all values are expressed as the mean ± SD.

*^#^Paralysis of the lower extremities indicates the muscle strength of the upper extremities*.

### Statistical Analysis

The chi-square test, Kruskal–Wallis test, Mann–Whitney test, Fisher test, and one-way ANOVA were used to test for statistically significant differences. Long-term follow-up outcomes were analyzed by the Kaplan–Meier method in RStudio version 1.3 to determine the rates of patients with and without postoperative clinical recurrence. For statistical analyses, the software packages Prism version 7.0 and SPSS version 25.0 were used.

## Results

The clinical characteristics of the cases are presented in Table 1. None of the patients in the CM or revision group had a history of atlantoaxial dislocation. Two patients in the CM group suffered from dorsal kyphosis. In one patient in the CM group, syringomyelia progressed to the medulla oblongata acutely and was partially relieved after FMMD (8). Interestingly, the preoperative neutrophil-to-lymphocyte ratio (NLR) of the patient was as high as 6.5. Most patients in the subarachnoid compression group suffered a history of trauma; among them, one had local subarachnoid adhesions.

### Comparison of Neurological Markers

The CM group comprised 106 patients (mean age, 48.0 ± 12.7). The revision group (47.0 ± 11.3) was similar to the CM group. Most patients in all three groups were in the range of 40–60 years old, but at our center, pediatric patients are frequently treated; thus, the number of patients in the CM group aged 1–20 years in this study was relatively low. The PTS patients were mostly male (*p* < 0.0001), and there was no significant difference in age compared with the other groups (*p* = 0.8018). Nearly half of the PTS group had experienced a complete SCI. The interval after injury was longer in the PTS group than in the revision group (*p* = 0.0004), but the natural history of syringomyelia was shorter (*p* = 0.0173). The initial symptoms of syringomyelia were usually paraesthesia (*p* = 0.258) and neuropathic pain (13.33%), but these symptoms were rare in the PTS group. The symptoms in the PTS group were mainly hypoesthesia (*p* = 0.006) and abnormal muscle strength (*p* = 0.004), gait (*p* < 0.0001), and urination (*p* < 0.0001). Compared with the other groups, the revision group had a higher rate of occipital pain (*p* = 0.099) and swallowing dysfunction (*p* = 0.01), while differences in neuropathic pain (*p* = 0.178) and dysesthesia (*p* = 0.303) showed no significance.

The cavities in the PTS group were primarily located at the thoracolumbar level, while those in the craniocervical junction (CCJ) group were at the cervical-thoracic segment at the CCJ. The tension in the revision group was more than 75% (*p* = 0.009).

### Comparison of Biomarkers

SCI associated with PTS was more severe than that associated with CCJ. Compared with that in the PTS group, the SCI caused by syringomyelia associated with the CCJ was more distributed in grade D (*p* = 0.003). Moreover, the decrease in pinprick and light touch sensation was higher in the PTS group (*p* = 0.0005, *p* < 0.0001, respectively). However, the SCI history in the PTS group often caused irreversible damage to SC function. Although the history in the revision group was longer, there was no significant difference in the ASIA score between the revision and CM groups. There was no significant difference in upper extremity muscle strength among the three groups (*p* = 0.1012). It should be noted that the previous SCI in PTS usually does not affect upper extremity muscle strength. However, the subdural adhesions were often worse (*p* < 0.0001) in the PTS group. Additionally, there were no significant differences among the groups in PBIMs except for in the red blood cell (RBC) count, which showed marginal statistical significance (*p* = 0.0421), presumably because the blood–SC barrier limited the reflection of the difference in chronic inflammation or the sample size was too small.

### Comparison of Surgical Prognosis

Compared with the CM group, the revision group had a higher proportion of cerebellar tonsil manipulation, but there was no significant difference (*p* = 0.276). The PTS group had the highest rate of adhesion lysis, followed by fusion. After FMMD with or without revision, complications within 7 days were observed in 23.07 and 9.43% of patients, respectively, without a significant difference for patients with PTS (*p* = 0.133). The degree of syringomyelia decreased at 58.8% of the CM group and remained stable in 39.95%. The rate of postoperative increase was low, at 1.25%. Due to the higher tension of the syrinx in patients in the revision and PTS groups, the rate of syrinx cavity decrease was higher in this group, but the difference did not reach statistical significance (*p* = 0.123). The analysis of long-term outcomes suggested no significant differences among the CM, revision, and PTS groups (*p* = 0.257) (Figure 2).

Due to the influence of intradural manipulation, the rate of headache relief was low in the short term, but the rate of improvement was higher in the CM group after 3 months (70 vs. 41.6%). In terms of neurogenic pain, the improvement rate was higher in the CM and revision groups than that in the PTS group (55.2%, 52.6 *vs*. 42.9%). In terms of paraesthesia, the improvement rate was higher in the CM and PTS groups than in the revision group (67.5%, 58.3 vs. 45.4%). Regarding hypoesthesia, the improvement rate was higher in the PTS group than in the CCJ and revision groups (53.8 vs. 33.3%, 45.4%). Furthermore, because of the longer history in the revision group, the symptoms of those patients were often more severe. Although the lower limb symptoms in the PTS group were more severe in terms of muscle strength, the improvement rate of muscle strength related to cavitation was slightly lower in the PTS group than in the CCJ and revision groups (38.5 vs. 40%, 38.9%). In terms of gait, the improvement rate was higher in the CM group than in the revision and PTS groups (54.8 vs. 28.5%, 23.1%), but the history of trauma in the PTS group can easily lead to residual gait disorder, which prevents the exclusion of confounding factors. In terms of urination, the improvement rate was higher in the revision and PTS groups than in the CM group (66.7%, 40 vs. 33.3%), but the proportion of patients with urination disorders was relatively low in the CM and revision groups. The CCJ group had a higher rate of cranial nerve symptom remission among those treated with posterior decompression (72.7, 66.6%), while in the PTS group; there were 2 cases of bulbar cavity-causing related symptoms, 1 of which was relieved after surgery. In terms of sweating symptoms, the remission rate was lower in the CM group (14.3, 0%); there were two patients with sweating symptoms in the PTS group, 1 of whom achieved relief after surgery.

## Discussion

With the aging of society, an increasing number of cervical degenerative disease patients experience central SCI, many of whom have symptoms that are more severe in the upper limbs than in the lower limbs (17). Syringomyelia is an expansion of the central canal of the SC, which is the simplest form of central SCI. With the help of a syringomyelia model, central SCI caused by various conditions can be better studied. CM is the most common clinical cause of syringomyelia, and spinal obstruction-related syringomyelia is similar to that caused by compression (14). However, the long-term natural history of syringomyelia remains unclear (8, 18). In this study, the history of syringomyelia related to the CCJ, especially in the revision group, was significantly longer than that in the PTS group. We suspect that the duration of the natural history may be related to the extent of SCI. In addition, most patients in these three groups underwent intradural decompression. Therefore, we made relevant clinical comparisons of the similarities and differences among the three groups to improve our understanding of central SCI. In future studies, we will explore the underlying molecular mechanism, deepen our understanding of the role of ependymal cells in SCI repair, and provide a theoretical basis for endogenous SC repair.

### Natural History in Different Subgroups

In our study, the patients with PTS were mostly male. This might be because men are more likely to be injured. Studies have pointed out that the fluid in the cavity mainly comes from the subarachnoid space (19). In the case of abnormal CSF pulsation, the fluid can enter the central canal of the SC through the perivascular space. In our study, the proportion of patients with high-tension cavitation was higher in the PTS group than that in the CM group, but the data of the revision group may be influenced by outpatient selection bias. One potential cause for this difference is that the mechanisms of the formation of and postoperative changes in the cavity are different in the PTS and CM groups, such as the presence of more serious subdural adhesions and greater blood–brain barrier destruction in PTS (20). However, some authors have suggested that there is no correlation between tension and injury (21). With the expansion of the central canal, SCI and related dysfunction gradually became aggravated, which is also in line with the clinical cavities in the CM group. In the CM group, the history of related symptoms was longer than that in the PTS group, and the progression of SCI was slower. Generally, pain-temperature cross-fibers immediately in front of the central canal are the first to be involved, and the typical clinical manifestations of segmental pain-temperature sensation and tactile separation appear. While our data showed no significant differences in upper limb muscle strength among the three groups, the proportion of patients with hypoesthesia was higher in the revision and PTS groups. In addition, we noticed that both pain and light sensation often declined in the PTS group, while a higher proportion of patients with dissociated sensory loss was observed in the CM group, which suggests that PTS is associated with more severe trauma. However, neurogenic pain was rare in the PTS group, which suggests that central canal dilatation was more likely to be accompanied by SC parenchyma damage in the PTS group. These findings in the PTS group may be related to the faster progression of abnormal CSF circulation dysfunction; further enlargement of the central canal involves the anterior horn neurons and manifestations such as muscle atrophy. In clinical practice, we also noticed that in the CCJ group, atrophy of the first and second interosseous muscles of the upper limb or small interosseous muscles and ulnar finger extension difficulty were common. However, due to the short medical history, patients in the PTS group seldom showed anterior motor horn injury of the upper limb or muscle atrophy. Longitudinal conduction tracts farther away from the central canal, such as the corticospinal tract and spinothalamic tract, always show signs of damage in the later stages of the disease. The proportion of patients with impaired motor function and gait was higher in the revision group than in the CM group, which also supports this view. However, many studies have found that the size of the syrinx in syringomyelia is not related to the severity of clinical symptoms (21, 22). Our previous basic research found that SCI and changes occurred in the early stage of cavity formation. The occurrence of SCI caused by cavities may not be solely due to central canal expansion, but it is likely that central canal expansion and SCI coexist (14, 20). Therefore, it is necessary to further clarify the pathological damage and the mechanism of SCI caused by syringomyelia.

### Biomarkers

Generally, it has been suggested that immunity and inflammation play major roles in the initiation and development of pancreatic cancer, hepatocellular carcinoma, glioma, and other tumors (23–25). It has been shown that inflammation is related to changes in peripheral blood leukocytes that are related to the NLR (26). The preoperative levels of PBIMs, for example, neutrophils, lymphocytes, monocytes, or their ratios, have been suggested to be related to the prognosis and outcomes of immunotherapy in patients with some types of cancer (27–29). Ependymal cells are activated after SCI and then play a reparative role *via* cell proliferation, differentiation, and migration (30). In the previous studies, we found that the number of ependymal cells increased significantly after the formation of syringomyelia. In addition, syringomyelia was found to be significantly related to inflammatory pathways through SC tissue transcriptomic and metabolomic analysis in a syringomyelia animal model (31). The present study confirmed the negative predictive value of leukocyte counts and the NLR for inflammation. However, we noticed that there was a patient with acute syringomyelia progression in the CM group with a lumbar compression fracture, and his NLR was as high as 6.5, which may indicate that the inflammatory reaction in the acute phase was more severe (8).

Some researchers have suggested that oxidative stress plays an important role in the pathophysiology of both acute and chronic SCI (32, 33). Erythrocytes have been shown to have potential as markers for the diagnosis of some diseases (34). Some authors have suggested that erythrocytes lose all of their organelles when they mature, causing a reduction in their potential to replace proteins that have lost their functions, which make them prone to any aberrations and very sensitive to oxidative stress (35). Wozniak suggested that higher lipid peroxidation levels will increase the concentrations of thiobarbituric acid-reactive substances in the RBCs of cervical SCI patients (36). Recent studies have suggested erythrocytes as a potential biomarker in the treatment of oxidative stress-associated diseases, such as chronic obstructive pulmonary disease, cardiorespiratory fitness in chronic SCI individuals (37), and primary open-angle glaucoma (38). Additionally, some authors have already shown that mild anemia or low RBC levels can be found after SCI (39). However, other authors have reported abnormally low levels of RBCs in early chronic SCI patients and augmentations over time, with the RBC count, hemoglobin, and hematocrit returning to near normal levels in late chronic SCI patients (40). Further research is needed for elucidation of the molecular mechanism of SCI in syringomyelia.

## Conclusions

PTS tends to progress faster than CM. The first symptom is usually paraesthesia, and SCI in PTS is more serious than syringomyelia associated with the CCJ. The inflammation related to syringomyelia caused by different etiologies cannot be distinguished through PBIMs except for the RBC count.

## Data Availability Statement

The raw data supporting the conclusions of this article will be made available by the authors, without undue reservation.

## Author Contributions

CY: writing—original and draft data curation. JG: writing—reviewing, editing, and visualization. YD, ZF, XW, and QY: software and draft data curation. CZ: software. SJ, KW, WD, HW, and ZC: resources. ZL: methodology and resources. XW and ZW: resources and visualization. FJ: writing—reviewing and editing and project administration. All authors contributed to the article and approved the submitted version.

## Funding

This study was supported by Beijing Municipal Science and Technology Commission (Grant number: Z191199996619048) and Beijing Municipal Commission of Education (Grant numbers: KZ202010025043 and 1192070315).

## Conflict of Interest

The authors declare that the research was conducted in the absence of any commercial or financial relationships that could be construed as a potential conflict of interest.

## Publisher's Note

All claims expressed in this article are solely those of the authors and do not necessarily represent those of their affiliated organizations, or those of the publisher, the editors and the reviewers. Any product that may be evaluated in this article, or claim that may be made by its manufacturer, is not guaranteed or endorsed by the publisher.
