# Supplementary material for: Spinal Obstruction-Related vs. Craniocervical Junction-Related Syringomyelia: A Comparative Study
Source: Front Neurol. 2022 Aug 1;13:900441. doi: 10.3389/fneur.2022.900441 (PMC9376629; doi:10.3389/fneur.2022.900441)
Supplement: Supplementary file 1 [file Table_1.docx]

Sup Table 1: Changes in related symptoms of syringomyelia caused by different aetiologies

|  | Chiari I malformation  (n=106) | | | | Revision  (n=26) | | | | PTS  (n=15) | | | | **P**^$^ |
| --- | --- | --- | --- | --- | --- | --- | --- | --- | --- | --- | --- | --- | --- |
|  | Re | Im | Uc | Wo | Re | Im | Uc | Wo | Re | Im | Uc | Wo |  |
| **Headache^*^** | 13(43.3%) | 8(26.7%) | 9(30.0%) | 0 | 4(33.3%) | 1(8.3%) | 7(58.4%) | 0 | 0 | 0 | 0 | 0 | 0.158 |
| **Pain^*^** | 15(25.9%) | 17(29.3%) | 22(37.9%) | 4(6.9%) | 3(15.8%) | 7(36.8%) | 8(42.1%) | 1(5.3%) | 0 | 3(42.9%) | 4(57.1%) | 0 | 0.927 |
| **Dysesthesia^*^** | 14(18.9%) | 36(48.6%) | 20(27.0%) | 4(5.4%) | 1(4.5%) | 9(40.9%) | 9(40.9%) | 3(13.7%) | 0 | 7(58.3%) | 5(41.7%) | 0 | 0.232 |
| **Sensory^*^** | 2(3.3%) | 18(30%) | 37(61.7%) | 3(5.0%) | 0 | 10(45.4%) | 10(45.4%) | 2(9.2%) | 0 | 7(53.8%) | 6(46.2%) | 0 | 0.432 |
| **Motor** | 10(20%) | 10(20%) | 29(58%) | 1(2%) | 2(11.1%) | 5(27.8%) | 10(55.5%) | 1(5.6%) | 1(7.7%) ^#^ | 4(30.8%) | 7(53.8%) | 1(7.7%) | 0.484 |
| **Gait** | 9(29%) | 8(25.8%) | 12(38.7%) | 2(6.5%) | 1(7.1%) | 3(21.4%) | 9(64.4%) | 1(7.1%) | 0 | 3(23.1%) | 10(76.9%) | 0 | 0.127 |
| **Sphincter function** | 0 | 3(33.3%) | 6(66.7%) | 0 | 0 | 2(66.7%) | 1(33.2%) | 0 | 0 | 4(40%) | 6(60%) | 0 | 0.713 |
| **Cranial nerve** | 5(45.4%) | 3(27.3%) | 3(27.3%) | 0 | 3(33.3%) | 3(33.3%) | 2(22.2%) | 1(11.1%) | 0 | 1(50%) | 1(50%) | 0 | 0.828 |
| **Sweating** | 0 | 2(14.3%) | 12(85.7%) | 0 | 0 | 0 | 2(100%) | 0 | 0 | 1(50%) | 1(50%) | 0 | 0.554 |

Re: Recovery; Im: Improved; Uc: Unchanged; Wo: Worsen.

^*^One patient was excluded because of cerebral infraction.

^#^upper extremities was improved.

^$^Fisher exact test.
