# Supplementary material for: Spinal Obstruction-Related vs. Craniocervical Junction-Related Syringomyelia: A Comparative Study
Source: Front Neurol. 2022 Aug 1;13:900441. doi: 10.3389/fneur.2022.900441 (PMC9376629; doi:10.3389/fneur.2022.900441)
Supplement: Supplementary file 2 [file Table_2.docx]

Sup Table 2: Comparison of preoperative complete blood counts of syringomyelia caused by different aetiologies

| **Laboratory testing** | CMI  (n=106) | Revision  (n=26) | PTS  (n=15) | P |
| --- | --- | --- | --- | --- |
| **WBC** | 6.1±1.6 | 5.6±1.5 | 6.1±1.6 | 0.34^#^ |
| **RBC** | 4.5±0.5 | 4.5±0.6 | 4.8±0.4 | **0.0421**^#^ |
| **Hb** | 134.6±14.3 | 132.4±16.9 | 142.6±10.5 | 0.0622^#^ |
| **Pct** | 40.3±3.9 | 39.7±4.7 | 42.2±3.1 | 0.1192^#^ |
| **MCV** | 89.8±5.5 | 89.1±7.2 | 88.2±3.4 | 0.4684^#^ |
| **MCH** | 30.0±2.1 | 29.7±2.5 | 29.8±1.1 | 0.7878^#^ |
| **MCHC** | 333.5±9.7 | 332.7±11.2 | 337.9±8.7 | 0.1720^#^ |
| **RDW** | 12.9±0.9 | 12.9±0.8 | 12.9±0.7 | 0.9942^#^ |
| **PLT** | 251.0±62.0 | 237.7±50.8 | 232.0±73.1 | 0.1144^#^ |
| **N** | 3.6±1.3 | 3.2±1.1 | 3.7±1.0 | 0.3605^#^ |
| **L** | 2.0±0.6 | 2.0±0.5 | 1.9±0.5 | 0.7017^#^ |
| **MONO** | 0.4±0.1 | 0.3±0.1 | 0.4±0.1 | 0.3994^#^ |
| **EO** | 0.1±0.1 | 0.1±0.1 | 0.1±0.1 | 0.8045^#^ |
| **BA** | 0.03±0.02 | 0.02±0.02 | 0.06±0.08 | 0.1653^$^ |
| **NLR** | 1.9±1.0 | 1.7±0.4 | 2.0±0.7 | 0.4258^#^ |
| **PLR** | 131.3±42.8 | 125.9±28.8 | 121.0±25.0 | 0.4065^#^ |
| **MLR** | 0.2±0.09 | 0.2±0.03 | 0.2±0.05 | 0.3251^#^ |

^#^One-way ANOVA test.

^$^Kruskal-Wallis test.
