# Supplementary material for: Spinal Obstruction-Related vs. Craniocervical Junction-Related Syringomyelia: A Comparative Study
Source: Front Neurol. 2022 Aug 1;13:900441. doi: 10.3389/fneur.2022.900441 (PMC9376629; doi:10.3389/fneur.2022.900441)
Supplement: Supplementary file 3 [file Table_3.docx]

Sup Table 3: Changes in the size of syringomyelia caused by different aetiologies before and after the operation.

| **Grade** | Chiari I malformation  (n=106) | | Revision  (n=26) | | PTS  (n=15) | |
| --- | --- | --- | --- | --- | --- | --- |
|  | Pre | Follow-up^*^ | Pre | Follow-up | Pre | Follow-up |
| **A** | 51(48.1%) | 9(11.3%) | 19(73.1%) | 1(3.8%) | 11(73.4%) | 1(6.7%) |
| **B** | 24(22.6%) | 31(38.7%) | 6(23.1%) | 16(61.5%) | 2(13.3%) | 7(33.3%) |
| **C** | 26(24.5%) | 34(42.5%) | 1(3.8%) | 9(34.7%) | 2(13.3%) | 6(53.3%) |
| **D** | 5(4.7%) | 5(6.3%) | 0 | 0 | 0 | 1(6.7%) |
| **E** | 0 | 1(1.2%) | 0 | 0 | 0 | 0 |
| **Total improved** |  | 47(58.8%) |  | 19(73.1%) |  | 11(73.3%) |

^*Among them, 26 patients were followed up in a local hospital or without imaging follow-up.^
